# The Trichopterygini (Lepidoptera, Geometridae) of Austral South America: description of new species from Chile

**DOI:** 10.3897/zookeys.832.30851

**Published:** 2019-03-19

**Authors:** Mario I. Ramos-González, Carlos Zamora-Manzur, Dania Saladrigas Menés, Luis E. Parra1

**Affiliations:** 1 Departamento de Zoología, Facultad de Ciencias Naturales y Oceanográficas, Universidad de Concepción, Casilla 160-C, Concepción, Chile; 2 Departamento de Ecología, Facultad de Ciencias, Universidad Católica de la Santísima Concepción, Alonso de Rivera 2850, Concepción, Chile; 3 Programa de Doctorado en Sistemática y Biodiversidad, Facultad de Ciencias Naturales y Oceanográficas, Universidad de Concepción, Concepción, Chile

**Keywords:** Andean region, *
Aloba
*, *Butleriana, Fueguina*, *
Hoplosauris
*, Larentiinae, taxonomy, *
Warrenaria
*

## Abstract

Four new species belonging to the genera *Hoplosauris* Butler, *Butleriana* Parra, *Warrenaria* Parra, and *Fueguina* Parra from south-central Chile are described. The species are *H.morenoi* Ramos-González & Parra, **sp. n.**, *B.phoenix* Ramos-González & Parra, **sp. n.**, *W.onca* Ramos-González & Parra, **sp. n.**, and *F.araucana* Ramos-González & Parra, **sp. n.** The genus *Aloba* Warren is reassigned to tribe Trichopterygini and *A.carolinae* Ramos-González & Parra, **sp. n.** is described. Comparative diagnosis for all new species are provided, and illustrations of genitalia and the wing venation of the males for all new described species are given.

## Introduction

Geometridae is the second largest family within Lepidoptera, with approximately 23000 species worldwide (Scoble 1999; Scoble and Hausmann 2007; Van Nieukerken et al. 2011). More than 280 geometrid species are known from Chile, 252 of which are endemic (sensu Parra and Villagrán-Mella 2008). However, Parra (1995) estimated the diversity of Chilean geometrids to be at least 450 species.

Larentiinae is the second largest subfamily within Geometridae (Gaston et al. 1995; Scoble et al. 1995), its members occur in a wide variety of habitats, and is particularly abundant at great altitude in the tropics and in temperate forests (Holloway 1997), like those in south-central Chile (Hausmann and Parra 2009; Zamora-Manzur et al. 2011). Despite their high species-richness in Chile (i.e., around half of known Chilean geometrids are larentiines), there are relatively few studies related to these moths as compared to the Ennominae. So far, most research efforts focused on the revision of the genus *Eupithecia* Curtis (Vojnits 1985, 1992, 1994; Rindge 1987, 1991) and the tribe Trichopterygini (Parra 1991, 1996; Parra and Santos-Salas 1991, 1992; Parra et al. 2009, 2017).

Phylogenetically, Trichopterygini is a group at the base of Larentiinae, sister to all other larentiines, along with Chesiadini and Dyspteridini (Viidalepp 2011; Sihvonen et al. 2011; Õunap et al. 2016). The characteristics that distinguish Trichopterygini are the reduced size of the anal area of male hindwing to a fold, crevice, vesicle, flap or lobe, and the presence of a sternal pouch that does not occlude the tympanal opening (Dugdale 1980; Parra et al. 2017). In Chile, there are 14 genera and 39 species of trichopterygines. A phylogenetic hypothesis at the genus level was formulated by Parra (1991) and Parra et al. (2017). Despite this, no information regarding the natural history of most species is available and there are several undescribed taxa. The aim of this article is to describe five new species for the Chilean fauna and reassign one genus to Trichopterygini.

## Methods

Specimens from the Museum of Zoology of the Universidad de Concepción, Chile (**MZUC-UCCC**) and Zoologische Staatssammlung München, Germany (**ZSM**) were examined, as well specimens from field surveys, which were collected using a UV light trap and net sweeping. Activity period (i.e., flight times) and geographic distribution were obtained from each specimen label. All species were assigned to biogeographic provinces proposed by Morrone (2015).

The Barcode Index Number (BIN) of each species is reported which was obtained from the BOLDSystems v4 database (Ratnasingham and Hebert 2007). BINs represent a species-level taxonomic registry of the animal kingdom based on the analysis of nucleotide variation patterns in the barcode region of the cytochrome c oxidase I (COI) gene (Ratnasingham and Hebert 2013). Genetic distances (when available) were calculated using the Kimura 2-parameter (K2P) distance model, using the analytical tools provided by BOLDSystems v4 platform. Intra-specific and inter-specific genetic distances were reported as maximum and minimum distances, respectively. This genetic information facilitates the species delimitation and form the basis of future phylogenetic works (Brehm 2015, 2018).

The generic assignment of new taxa is based primarily on male genitalia and hindwing venation, which are important characters for the delimitation of species and genera within Trichopterygini (Parra et al. 2017). Species descriptions were made based on external morphological characteristics and genital armature from males and in some cases females. Wing and genitalia slides were prepared according to Parra (1991). Nomenclature for genitalia and external characteristics follow Klots (1970) and Scoble (1995) respectively.

## Taxonomy

### Aloba

Taxon classificationAnimaliaLepidopteraGeometridae

Warren, 1895

Aloba Warren 1895: 105.

#### Type species.

*Hoplosauriscinereus* Bartlett-Calvert, 1893, by original designation.

#### Diagnosis.

Palpi short, slightly tilted up. Male: Hindwing subtriangular, valvae with brush-like setal tuft with accessory undulated individual bristles. Female: ductus bursae half the length of corpus bursae. Posterior third of corpus bursae with longitudinal striation; the remaining two thirds with microspines.

#### Redescription.

Antennae filiform in both sexes, but subapically broadened in males. Thorax and abdomen with brownish scales, varying in color from greyish to yellowish shades. Forewings with wide and dark antemedial and postmedial bands; apical spot subquadrate and discal spot always present. In males, hindwings are reduced, subtriangular and whitish; its apex can be extended or not, and there is no visible modification in anal margin. Wing venation in males: forewing with two accessory cells; hindwing with Sc+R_1_ and Rs+M_1_ separated, M_2_ is free and M_3_ and Cu_1_ are pedunculated near the angle of discal cell, which is polygonal and it extends for one third of wing surface. Tibial formula 0-2-4 in both sexes. Abdomen is longer and narrower in males than in females. Male genitalia: ensiform valvae with cucullus projected apically, setal tuft is brush-like with accessory and undulated individual bristles, juxta with sclerotized S-shaped lateral processes. Female genitalia: corpus bursae sub-pyriform with longitudinal striation on the posterior third; the remaining two-thirds with microspines on its surface.

### Aloba carolinae

Taxon classificationAnimaliaLepidopteraGeometridae

Ramos-González & Parra sp. n.

http://zoobank.org/7C4B292A-F1BE-4EA7-9572-688B20CE23A3

**Figures 1–8.**
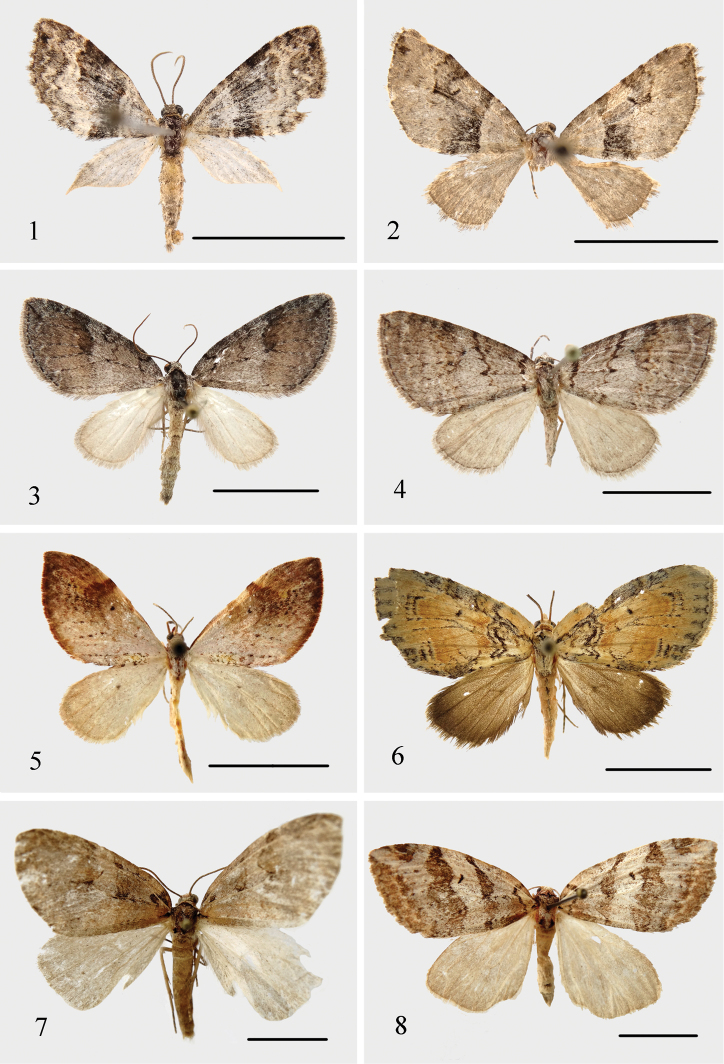
Adults. **1–2***Alobacarolinae* Ramos-González & Parra, sp. n. **1** male (holotype) **2** female (paratype). **3, 4***Hoplosaurismorenoi* Ramos-González & Parra, sp. n. **3** male (holotype) **4** female (allotype). **5***Butlerianaphoenix* Ramos-González & Parra, sp. n., male (holotype). **6***Warrenariaonca* Ramos-González & Parra, sp. n., male (holotype). **7, 8***Fueguinaaraucana* Ramos-González & Parra, sp. n. **7** male (holotype) **8** female (allotype). Scale bar: 1 cm.

**Figures 9, 10.**
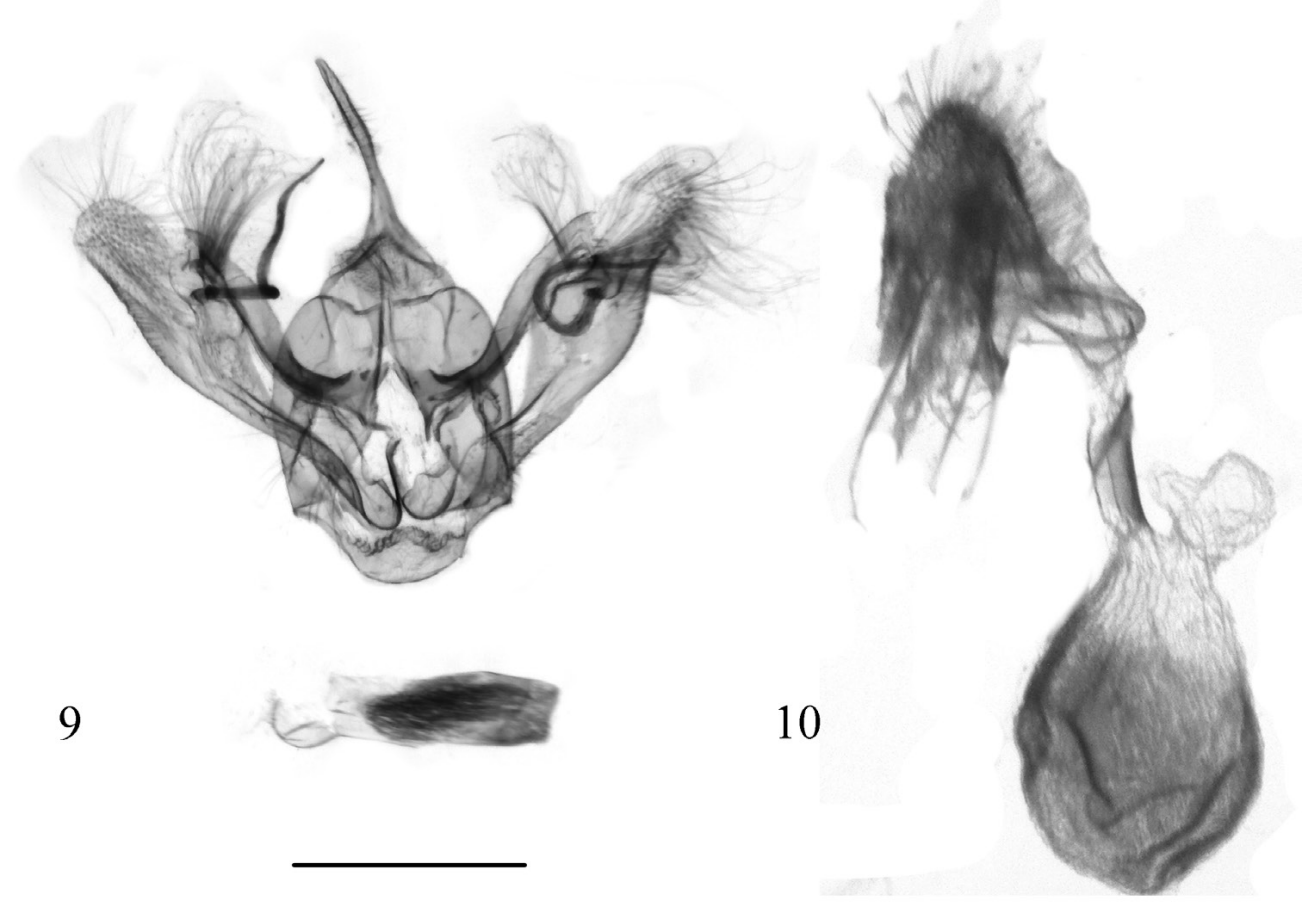
Genitalia of *Alobacarolinae* Ramos-González & Parra, sp. n. **9** male genitalia (paratype, MZUC-UCCC, slide No. FGCR LP 103) **10** female genitalia (allotype, MZUC-UCCC, slide no. FGCR LP 109). Scale bar: 1 mm.

Figures 1 , 2 , 9 , 10 , 17

#### Diagnosis.

This species is distinguished from *A.cinereus* (Bartlett-Calvert) by the following characteristics: saccus-vinculum broader, accessorial bristles in setal tuft apically undulated, and corpus bursae with the inner surface of its anterior half completely covered with microspines. Externally, this species stands out for its reduced wingspan and for the feather-like extended hindwing apex in males.

#### Description.

Male (Fig. 1). Head: antennae filiform, subapically broadened; palpi short, subequal to eye diameter and slightly tilted upwards. Thorax: Patagia and tegulae covered by piliform grayish scales. Forewings: background color ashy gray; termen rounded, with piliform ashy scales; basal band blackish brown; antemedial band slender, blackish brown, and surrounded by two stripes of ashy-white scales; medial band blackish brown, with a small and subrounded ashy spot on the costal third, medial band with proximal margin arcuate and distal margin with five undulations. Some specimens, in both sexes, with a blackish spot near half of the band and the subrounded ashy spot on the costal third is absent or located in the anal third; postmedial band slender blackish brown and surrounded with two stripes of ashy-white scales; subterminal band zigzagging of whitish scales; apical spot on the wing apex subquadrate and blackish. This spot connects with subterminal band; terminal band formed by a dashed stripe of short blackish spots; distal spot present and blackish. Hindwings: ashy-white, reduced, one-third the length of forewings, triangular with prolonged apex, anal margin with no visible modification; discal spot not visible. Wing venation in males (Fig. 17): same as the genus. Male genitalia (Fig. 9): valvae ensiform, cucullus apically projected, sclerotized costa, subapical setal tuft brush-like with thick, large and undulated individual accessory bristles; saccus subrounded; juxta with quadrate base and posterior apex indented, with two sclerotized and disjointed S-shaped lateral processes, which extend to the height of the transtilla; socius triangular; transtilla projected in a Y-shaped, with apices equal in length. Aedeagus tubular; cornuti arranged as a longitudinal group in the vesica. Female (Fig. 2). Similar to male, but with filiform antennae slighter and hindwings not reduced, quadrangular and ashy-grey. Female genitalia (Fig. 10): ductus bursae half the length of corpus bursae; corpus bursae membranous, subpyriform, with straight longitudinal striation that does not exceed one-third of corpus bursae; anterior region of corpus bursae with microspines on its entire inner surface; cestum present; posterior apophyses larger than anterior apophyses.

#### Type material.

Holotype: 1 ♂, pinned, Chile, Concepción, Fundo El Guindo Point 1A, 36°50.18’S, 73°1.40’W, 20-X-2014, leg. M. Ramos & C. Rose, “Holotype *Alobacarolinae*” [red handwritten label] (MZUC-UCCC); allotype: 1 ♀, pinned, with genitalia in microscope slide, Chile, Concepción, Fundo El Guindo Point 1B, 36°50.21’S, 73°1.39’W, 26-X-2014, leg. M. Ramos & C. Rose, “FGCR LP 109” [genitalia slide] “Allotype *Alobacarolinae*” [red handwritten label] (UCCC-MZUC).

Paratypes: 46 males, 5 females. Chile: **Curicó**: Los Queñes, 34°59.65’S, 70°48.78’W, 721 m, 10-II-2016, leg. M. Ramos & M. Astrosa (1 ♂) (MZUC-UCCC); P.N. Radal Siete Tazas, 35°28’S, 71°W, 1100 m, 19-XII-2000, leg. Gielis [ID BC ZSM Lep 07419, barcode sequence 658 bp; ID BC ZSM Lep 07433, barcode sequence 658 bp] (2 ♂) (ZSM). **Diguillín**: Termas de Chillán, 05/11-II-2010, leg. G. Moreno (3 ♂) (MZUC-UCCC); Las Trancas, 01/08-II-2011, leg. G. Moreno (1 ♂) (MZUC-UCCC); Las Trancas, 03/10-I-2011, leg. G. Moreno (3 ♂) (MZUC-UCCC); Las Trancas, 14/20-I-2012, leg. G. Moreno (1 ♂) (MZUC-UCCC); Las Trancas, 16-I-1996, leg. unknown (3 ♂, 1 ♀) (MZUC-UCCC); Las Trancas, 11-I-1996, leg. unknown (1 ♂) (MZUC-UCCC); Las Trancas, 12-I-2017, leg. P. Bocaz (4 ♂); Las Trancas, Cabañas Bordenieve (IX FH), 36°54.83’S, 71°29.69W, 1236 m, 13-I-2017, “Hoplo-005” [wing slide], leg. L. Parra, M. Ramos & C. Zamora-Manzur (1 ♂) (MZUC-UCCC); Las Trancas, 36°54’S, 71°28’W, 1400 m, 14-I-2001, leg. Gielis & Wolf [ID BC ZSM Lep 07435, barcode sequence 658 bp; ID BC ZSM Lep 07431, barcode sequence 658 bp; ID BC ZSM Lep 07417, barcode sequence 658 bp] (2 ♂, 1 ♀) (ZSM). **Concepción**: Concepción, 15-XII-1961 (1 ♂) (MZUC-UCCC); same as holotype but “FGCR LP 011”, “FGCR LP 012” and “FGCR LP 013” [genitalia slides] (4 ♂) (MZUC-UCCC); same as holotype but 26-X-2014, female with “FGCR LP 096” [genitalia slide] (1 ♂, 1 ♀) (MZUC-UCCC); same as allotype but “FGCR LP 101” [genitalia slide] (1 ♂) (MZUC-UCCC); same as holotype but 03-XI-2014 (4 ♂) (MZUC-UCCC); same as allotype but 03-XI-2014, “FGCR LP 133” [genitalia slide] and “AMLP 103” [wing slide] (1 ♂) (MZUC-UCCC); same as holotype but Point 1C 36°50.23’S, 73°1.39’W, 26-X-2014, “FGCR LP 103” [genitalia slide] and “AMLP 0088” [wing slide] (3 ♂) (MZUC-UCCC); same as holotype but Point 2A 36°50.23’S, 73°1.47’W, 21-X-2014, “FGCR LP 110”, “FGCR LP 132” [genitalia slide] and “AMLP 0102” [wing slide] (3 ♂) (MZUC-UCCC); same as holotype but Point 2A 36°50.23’S, 73°1.47’W, 17-XI-2014 (2 ♂) (MZUC-UCCC); Chiguayante, 06-III-2002, leg. P. Bocaz (1 ♀) (MZUC-UCCC). **Cautín**: 15 km NE from Colico Lake, 39°3’S, 71°49.02’W, 400 m, 03-XII-2000, leg. Gielis [ID BC ZSM Lep 03051, barcode sequence 613 bp] (1 ♂) (ZSM). **Palena**: Fiordo Comau, San Ignacio del Huinay, 42°22.82’S, 72°24.8’W, 35 m, 20-II-2008, leg. T. Roy [ID BC ZSM Lep 16933, barcode sequence 658 bp; ID BC ZSM Lep 16922, barcode sequence 658 bp; ID BC ZSM Lep 16936, barcode sequence 658 bp; ID BC ZSM Lep 16923, barcode sequence 658 bp] (3 ♂, 1 ♀) (ZSM).

#### Distribution.

This species occurs between Curicó and Palena provinces. It is distributed in parts of Santiago, Maule and Valdivian Forest biogeographic provinces, Central Chilean and Subantarctic subregions, Andean region.

#### Flight period.

Specimens were captured from October to March.

#### Molecular data.

BOLD:AAD7992. Ten available sequences of DNA barcode: BC ZSM Lep 07419 (Molina), BC ZSM Lep 07433 (Molina), BC ZSM Lep 07431 (Pinto), BC ZSM Lep 07417 (Pinto), BC ZSM Lep 07435 (Pinto), BC ZSM Lep 03051 (Cunco), BC ZSM Lep 16933 (Huinay), BC ZSM Lep 16922 (Huinay), BC ZSM Lep 16936 (Huinay), BC ZSM Lep 16923 (Huinay). Maximum intraspecific distance: 0.76%; Minimum genetic distance with *A.cinereus*: 9.35%.

#### Etymology.

The species name is dedicated to the collector and biologist Carolina Rose Garrido, Concepción, Chile.

### Hoplosauris morenoi

Taxon classificationAnimaliaLepidopteraGeometridae

Ramos-González & Parra sp. n.

http://zoobank.org/BCE65057-D13A-4829-80BA-C9E73B9EB258

**Figures 11, 12.**
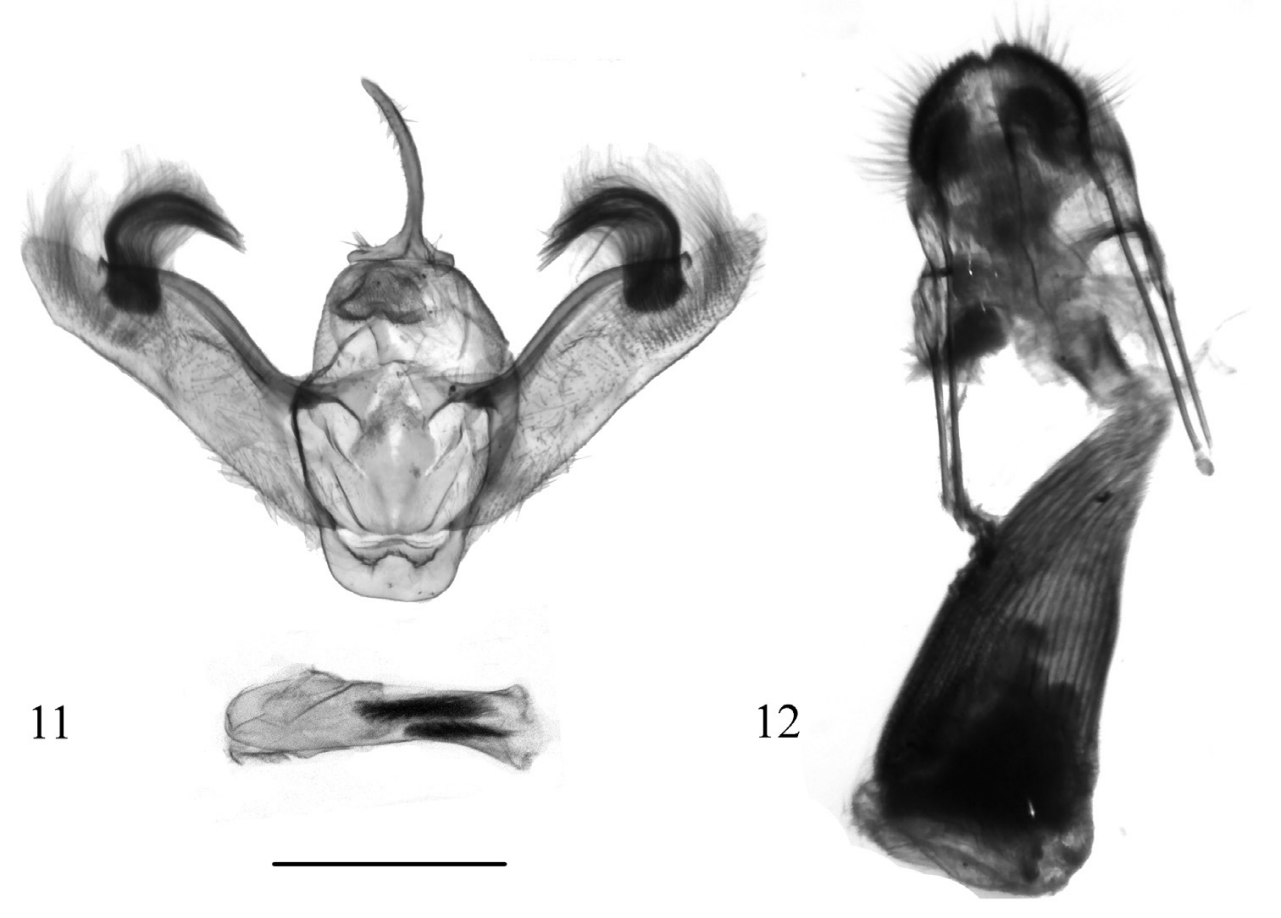
Genitalia of *Hoplosaurismorenoi* Ramos-González & Parra, sp. n. **11** male genitalia (paratype, MZUC-UCCC, slide No. AMLP 0300) **12** female genitalia (paratype, MZUC-UCCC, slide No. AMLP 0122). Scale bar: 1 mm.

Figures 3 , 4 , 11 , 12 , 18

#### Diagnosis.

This species and *H.heliconoides* Butler share the following characters: valvae with sclerotized costa and apically rounded; in females, two-thirds (or more) of corpus bursae with longitudinal striation. However, in the case of *H.morenoi* there are microspines on the sclerotized longitudinal striation only in the mid-ventral region (autapormorphy). The external morphology is highlighted by the grayish forewing, which is crossed by coppery-brown bands.

#### Description.

Male (Fig. 3). Head: antennae filiform, subapically broadened; palpi porrect and subequal to eye diameter. Thorax: Patagia and tegulae covered by piliform ashy and brown scales. Tibial formula 0-2-4. Forewings: background color dark gray; termen rounded, with piliform dark-gray scales; basal band straight coppery-brown; antemedial band coppery-brown, slightly zigzagging; postmedial band coppery-brown, straight, twice as wide as the basal and antemedial bands; subterminal band whitish, zigzagging; apical spot slender and blackish which connects with subterminal band; terminal band formed by a dashed stripe of short coppery-brown spots; discal spot present and blackish. Hindwings: reduced, half the length of forewings, subrounded, pale ashy, with no visible modification at the base of anal margin; without discal spot. Wing venation in males (Fig. 18): forewing with two accessory cells; hindwing with Sc+R_1_ and Rs connected by a weak transverse vein, one third before the end of the cell; Rs and M_1_ pedunculated; M_2_ free and M_3_ and Cu_1_ pedunculated; discal cell triangular and extends for a quarter of wing surface; anal cell present, formed by a weak transverse vein towards the middle of the discal cell that connects cubital stem with anal margin. Male genitalia (Fig. 11): valvae ensiform, costa sclerotized and rounded, cucullus apically extended, subapical setal tuft brush-like; saccus subrounded; juxta with subquadrangular base and indented posterior apex, with two disjointed lateral processes that have subtriangular apex, these processes extend to the height of the transtilla; uncus setose and curved; socius triangular; transtilla projected in a Y-shaped, with apices unequal in length. Aedeagus tubular; cornuti arranged as two longitudinal groups in the vesica. Female (Fig. 4). Similar to males but with filiform antennae slighter and hindwings not reduced, subquadrangular and pale ashy. Female genitalia (Fig. 12): ductus bursae one-sixth the length of corpus bursae; corpus bursae subpyriform, sclerotized, with straight longitudinal striations that exceed two-thirds of corpus bursae and mid-ventral region with rows of microspines; posterior apophyses larger than anterior apophyses.

#### Type material.

Holotype: 1 ♂, pinned, Chile, Icalma, 02-II-2017, leg. H. Torres, “Holotype *Hoplosaurismorenoi*” [red handwritten label] (MZUC-UCCC); allotype: 1 ♀, pinned, Chile, Malalcahuello, 20-I-2017, leg. C. Zamora-Manzur, “Allotype *Hoplosaurismorenoi*” [red handwritten label] (MZUC-UCCC).

Paratypes: 17 males, 7 females. Chile: **Diguillín**: Volcán Chillán, 03-III-1979, coll. light traps (1 ♂) (MZUC-UCCC); Las Trancas, 7-I-1987, leg. M. Beéche, “AMLP 0030” [wing slide] (1 ♂) (MZUC-UCCC); Las Trancas, 03/10-I-2011, leg. G. Moreno, “AMLP 0122” [female genitalia slide] (1 ♂, 3 ♀) (MZUC-UCCC); Las Trancas, 08-I-1996, leg. M. Beéche (1 ♂); Las Trancas, 16-I-1996, coll. Phototropic trap (1 ♂) (MZUC-UCCC); Las Trancas, 14/20-I-2012, leg. G. Moreno, “UCCC_MZUC_Lep_0388” [male ID code] (1 ♂, 1 ♀) (MZUC-UCCC). **Malleco**: Curacautín, 20-II-2008, leg. O. Vergara & J. Guzmán, “BC LP 0039” [Barcode voucher] (1 ♀) (MZUC-UCCC); same as holotype but 21-II-2017, “AMLP 0300” [genitalia slide] (1 ♂) (MZUC-UCCC); Curacautín, Río Blanco, 38°12’S, 71°55.99’W, 28-II-1995, leg. H. Thoeny [ID BC ZSM Lep 07781, barcode sequence 530 bp; ID BC ZSM Lep 07779, barcode sequence 570 bp; ID BC ZSM Lep 07628, barcode sequence 577 bp] (1 ♂, 2 ♀) (ZSM); Pino Hachado, 38°12’S, 71°55.99’W, 18-II-1995, leg. H. Thoeny [ID BC ZSM Lep 07634, barcode sequence 582 bp] (1 ♂) (ZSM); Contulmo, Palo botado, 02-II-1953, leg. L.E. Peña (1 ♂) (MZUC-UCCC). **Cautín**: Termas de Río Blanco, III-1951, leg. L.E. Peña (2 ♂) (MZUC-UCCC). **Coyhaique**: Laguna Azul, 23-I-2008, leg. L.E. Parra, “Genitalia 0258” [genitalia in microvial] (1 ♂) (MZUC-UCCC). **Capitán Prat**: Cochrane, Balsa Baker, 27-I-2008, “Genitalia 0245”, “Genitalia 0246”, “Genitalia 0257” [genitalia slides] leg. Muñoz-Escobar (4 ♂) (MZUC-UCCC).

#### Distribution.

This species occurs between Diguillín and Capitán Prat provinces. It is distributed in parts of Santiago, Maule and Valdivian Forest biogeographic provinces, Central Chilean and Subantarctic subregions, Andean region.

#### Flight period.

Specimens were captured from January to March.

#### Molecular data.

BOLD:AAH6701. Five available sequences of DNA barcode: BC LP 0039 (Curacautín), BC ZSM Lep 07781 (Curacautín), BC ZSM Lep 07779 (Curacautín), BC ZSM Lep 07628 (Curacautín), BC ZSM Lep 07634 (Lonquimay). Maximum intraspecific distance: 1.15%; Minimum genetic distance with *H.pachrophylloides* Parra: 7.74%.

#### Etymology.

The species name is dedicated to the naturalist and great collector Sr Guillermo Moreno Crisóstomo, Chillán, Chile.

### Butleriana phoenix

Taxon classificationAnimaliaLepidopteraGeometridae

Ramos-González & Parra sp. n.

http://zoobank.org/AE5952C6-72B0-423A-B065-2F0DA3EBEB2C

Figures 5 , 13 , 19

#### Diagnosis.

This species has a characteristic maculation pattern that easily distinguishes it from congeners: background color of forewings ashy-white, splashed with violaceous-red scales and crossed by dark violaceous-red antemedial and postmedial bands, which are more noticeable towards the costa. *Butlerianaphoenix* differs from *B.minor* (Butler, 1882), *B.oculata* (Mabille, 1885), *B.fumosa* (Butler, 1882), and *B.fasciata* (Butler, 1882) by the presence of free Rs and M1 veins on the hindwings of males. Additionally, *B.phoenix* shares with *B.fasciata* by having the A_1_ vein insinuated only at the base, but both species differ in male genitalia, as *B.phoenix* presents a strongly sclerotized costa, which exceeds the apex of cucullus, thereby forming a L-shaped notch at the apex of the valva.

#### Description.

**Figure 13.**
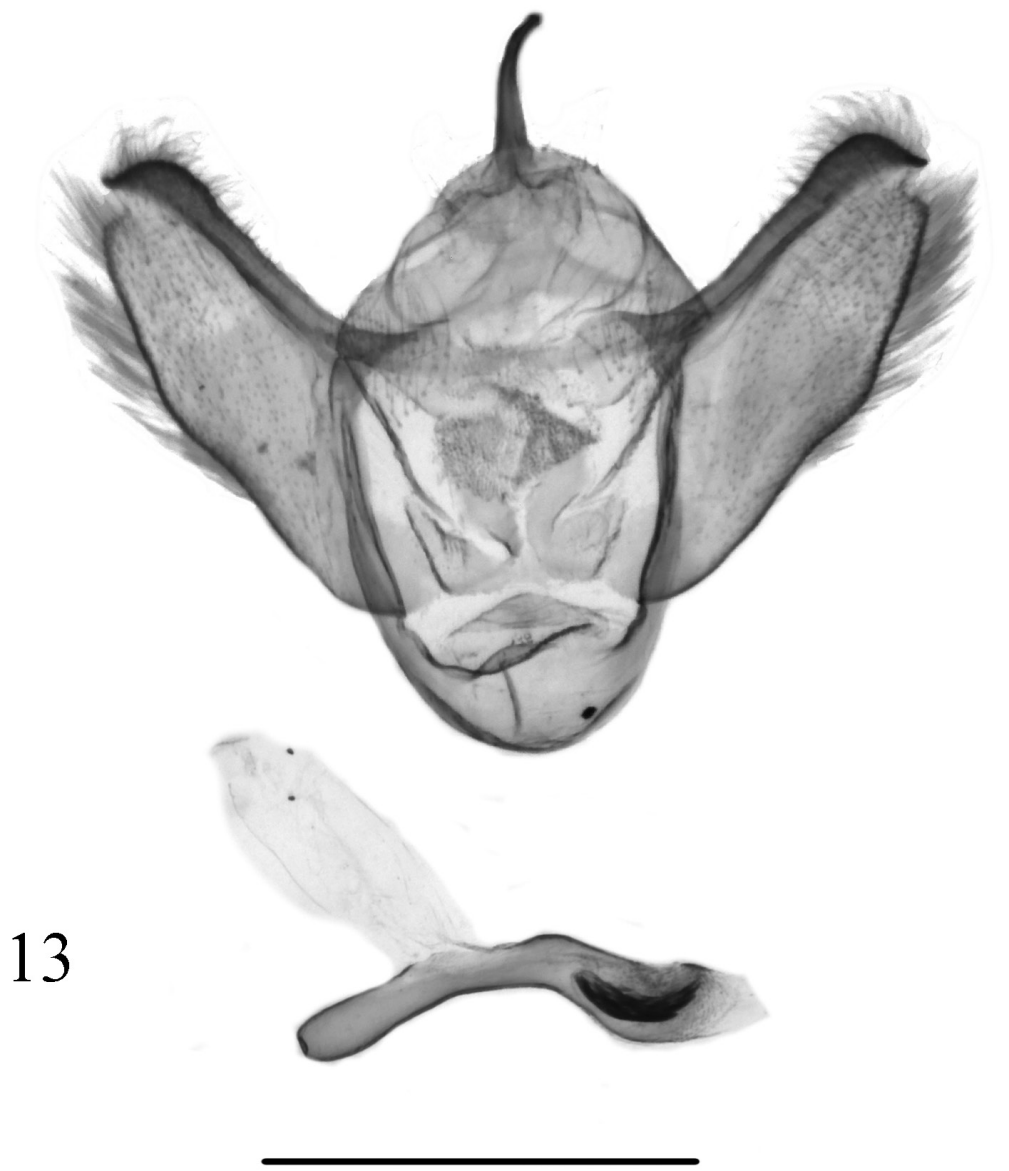
Male genitalia of *Butlerianaphoenix* Ramos-González & Parra, sp. n., male, holotype, MZUC-UCCC, slide No. AMLP 0141. Scale bar: 1 mm.

Male (Fig. 5). Head: antennae filiform, subapically broadened; palpi subequal to eye diameter, covered by erect piliform violaceous-red scales with third segment slightly curved down; frons covered with flattened reddish scales. Thorax: patagia covered by silvery-white and violaceous-red scales; tegulae covered by flattened scales, violaceous-red at proximal area and whitish towards its distal area. Tibial formula 0-2-4. Forewings: background color ashy-white, splashed with violaceous-red scales, with two irregular spots of golden-olive scales: one subapical the other in a post-basal position, on the anal margin; medial, cubital and anal veins framed by blackish scales, which are interspersed with the background color; termen rounded with piliform reddish scales; antemedial band dark violaceous-red, slightly arcuate; postmedial band dark violaceous-red, extended laterally towards the wing’s apex at the height of the two accessory cells; subterminal band diffuse, formed by two slender violaceous-red stripes; presence of an oblique blackish apical spot, which connects with postmedial band; discal spot blackish. Hindwings: reduced, three-quarters the length of forewings, subrounded, ashy-white, with an extended, narrow and subtriangular lobe at the base of the anal margin; discal spot blackish. Wing venation (Fig. 19): forewing with two accessory cells; hindwing with Sc+R_1_ and Rs anastomosed as far as one-third before the end of radial trunk; Rs, M_1_, M_2_, M_3_, Cu_1_ and Cu_2_ are free and located on the vertices of discal cell; Rs closer to M_1_ than to Sc+R_1_; M_2_ closer to M_3_; Cu_2_ originating from the middle of cubital trunk; in anal lobe only with A_2_ present, which is curved; A_1_ only insinuated at base of lobe; discal cell polygonal and it is extended for half of wing surface. Male genitalia (Fig. 13): valvae subrectangular, with a bulbous projection in the central area of anterior edge, costa strongly sclerotized, exceeding the apex of cucullus, apical notch L-shaped; saccus subrounded; juxta with subquadrangular base and forked posterior apex also with two lateral processes having a setose subtriangular apex connected each other in the midventral region, at the height of transtilla; uncus glabrous and curved; transtilla simple. Aedeagus tubular; cornuti arranged as a longitudinal group in the vesica. Female unknown.

#### Type material.

Holotype: 1 ♂, pinned, Chile, Chiloé, Quellón, 21-II-1951, leg. J.C. Vargas, “Museo”, “AMLP 0141” [genitalia slide] “Holotype *Butlerianaphoenix*” [red handwritten label] (MZUC-UCCC).

Paratypes: 4 males. Chile: **Chiloé**: Mocopulli, Ruta 5 Sur km 1170, 42°22.08’S, 73°43.73’W, 182 m, 03-II-2017, leg. M. Ramos-G, M. Ramos-SM & C. Rose (1 ♂) (MZUC-UCCC); Ancud, Pauldeo, 23-I-2005, “Colección Numhauser 2013”, “AMLP 0100” [wing slide], leg. Numhauser (1 ♂) (MZUC-UCCC). **Palena**: Fiordo Comau, San Ignacio del Huinay, pasture, 42°22.8’S, 72°24.78’W, 35 m, 04-I-2008, leg. A. Hausmann (1 ♂) [ID BC ZSM Lep 11682, barcode sequence 658 bp] (ZSM); Fiordo Comau, San Ignacio del Huinay, buildings, 42°22.86’S, 72°24.9’W, 20 m, 09-I-2008, leg. A. Hausmann, T. Greifenstein & L. Parra [ID BC ZSM Lep 11236, barcode sequence 632 bp] (1 ♂) (ZSM).

#### Distribution.

This species occurs in Chiloé and Palena provinces. It is distributed in a part of the Valdivian Forest biogeographic province, Subantarctic subregion, Andean region.

#### Flight period.

Specimens were captured from January to March.

#### Molecular data.

BOLD:AAD7597. Two available sequences of DNA barcode: BC ZSM Lep 11682 (Huinay), BC ZSM Lep 11236 (Huinay). Maximum intraspecific distance: 0.79%; Minimum genetic distance with *B.minor*: 10.59%.

#### Etymology.

The species name is a noun in the apposition, referring to the Phoenix (a mythical firebird), for the red/purple that is present in the moth’s forewing coloration pattern.

### Warrenaria onca

Taxon classificationAnimaliaLepidopteraGeometridae

Ramos-González & Parra sp. n.

http://zoobank.org/D02F7D34-4754-437E-9BB5-7E49B2539D20

Figures 6 , 14 , 20

#### Diagnosis.

This species can be easily distinguished from *W.martha* (Butler) by the presence of ashy-brown forewings, with less evident antemedial and postmedial bands, which have a ferruginous tone. Both species have an U-shaped posterior apex of the juxta in male genitalia but differs in the shape of the juxta’s base: subquadrangular in *Warrenariaonca* but subtriangular in *W.martha*.

#### Description.

**Figure 14.**
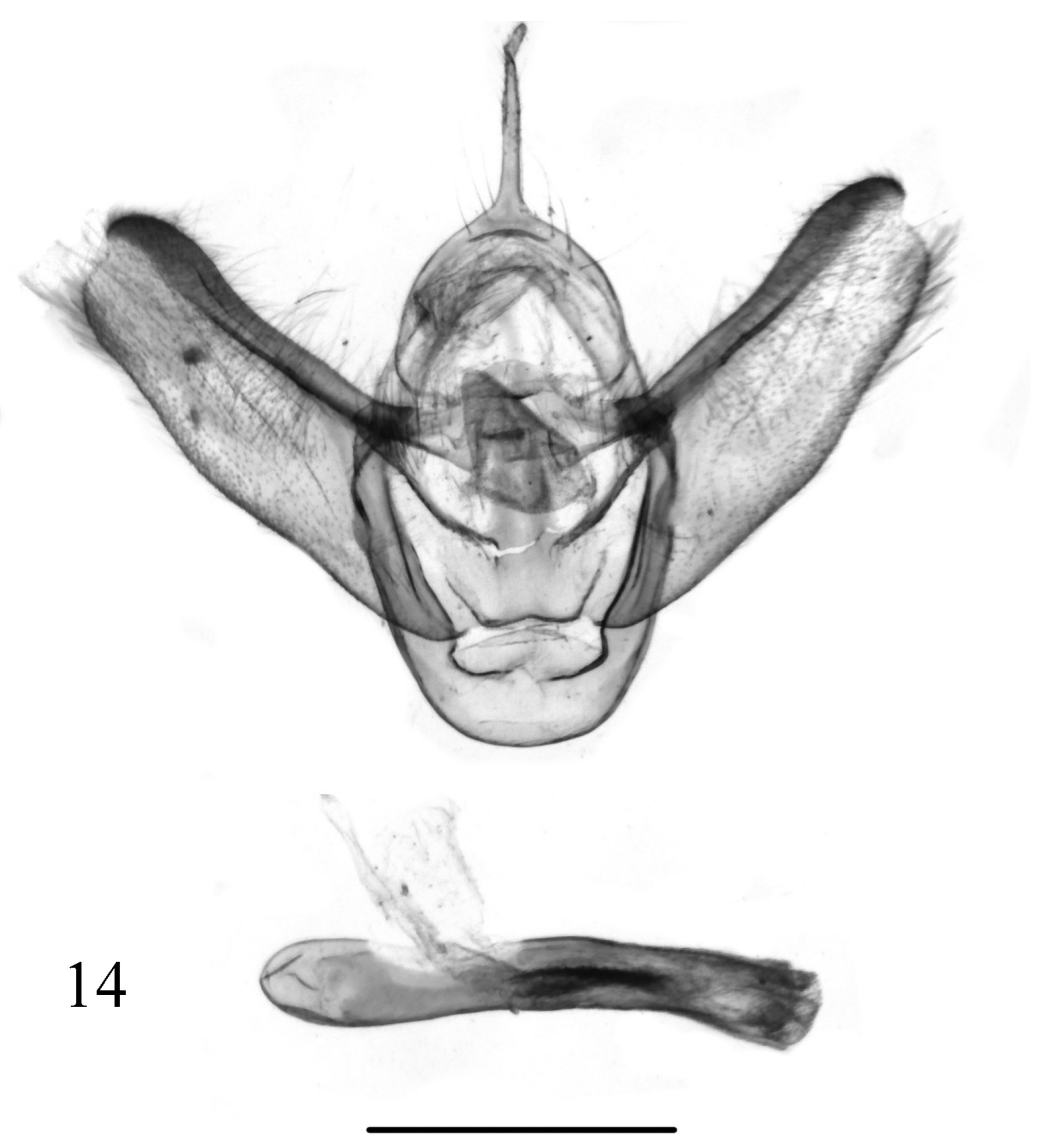
Male genitalia of *Warrenariaonca* Ramos-González & Parra, sp. n., male, holotype, MZUC-UCCC, slide No. AMLP 0137. Scale bar: 1 mm.

Male (Fig. 6). Head: antennae filiform, subapically broadened; palpi twice as long as eye diameter, covered by piliform straight light-brown scales; frons covered with imbricated flattened ashy-brown scales. Thorax: patagia covered by juxtaposed flattened ashy-brown scales; tegulae covered by piliform whitish, blackish and ashy-brown scales. Tibial formula 0-2-4. Forewings: background color ashy-brown splashed with blackish scales, slightly darker and with olivaceous tinge towards the costa and termen; M_3_ and Cu_1_ framed by blackish scales that cross the postmedial band; termen rounded, with dark piliform olivaceous-brown scales; basal region crossed by three wavy subcircular lines: proximal line light brown and diffuse, distal lines blackish and better defined than proximal one; costal margin of basal region only with a small subquadrate blackish spot, splashed with ferruginous-orange scales; antemedial band ferruginous-orange, slightly diffuse, zigzagging; postmedial band wavy, diffuse and composed of three slender ferruginous-orange stripes; costa of medial region mottled with blackish scales; subterminal band formed by two interrupted slender blackish stripes; adterminal band formed by rectangular interveinal spots; terminal band formed by blackish semicircles that are weakly connected with adterminal band; discal spot present and blackish. Hindwings: reduced, three-quarters the length of forewings, subrounded, dark brown, with an extended and subrounded lobe at the base of anal margin; discal spot blackish. Wing venation (Fig. 20): forewing with two accessory cells; hindwing with Sc+R_1_ and Rs linked by a transverse vein a quarter before of the end of the cell; Rs, M_1_, M_2_, M_3_ are free and located on the vertices of discal cell; Cu_1_ slightly arched, near the angle of cell; Cu_2_ inconspicuos, one-fifth before the angle of the cell; lobe crossed by sub-straight A_1_ and curved A_2_; discal cell polygonal and extend for half of wing surface. Male genitalia (Fig. 14): valvae subrectangular, costa strongly sclerotized, rounded apical notch with a small indention, about 1/16 the length of valvae; saccus subquadrate; juxta with subquadrangular base and U-shaped posterior apex, with two lateral processes that have a setose triangular apex and are connected in the midventral region, at height of transtilla; uncus simple and slightly setose; transtilla simple. Aedeagus tubular; cornuti arranged as two longitudinal groups in the vesica. Female unknown.

#### Type material.

Holotype: 1 ♂, pinned, Chile, Nahuelbuta, Río Picoiquen, 22-XII-1965, leg. Fetis, “AMLP 0137” [genitalia slide], “Holotype *Warrenariaonca*” [red handwritten label] (MZUC-UCCC).

#### Distribution.

This species is only known from the type locality: Chile, Araucanía, Malleco, Angol, Nahuelbuta, Río Picoiquen. This locality belongs to Maule biogeographic province, Central Chilean subregion, Andean region.

#### Flight period.

The single specimen was captured in December.

#### Etymology.

The species name is a noun in apposition and is in reference to the jaguar (*Pantheraonca*), a feline that inhabited the forests of southern South America until the end of the 19^th^ century and which gives its name to the type locality (Nahuelbuta) in Mapudungun language (*nawel*: jaguar; *füta*: big).

### Fueguina araucana

Taxon classificationAnimaliaLepidopteraGeometridae

Ramos-González & Parra sp. n.

http://zoobank.org/EA416114-32CA-4D92-BCEB-2F15171006F8

**Figures 15, 16.**
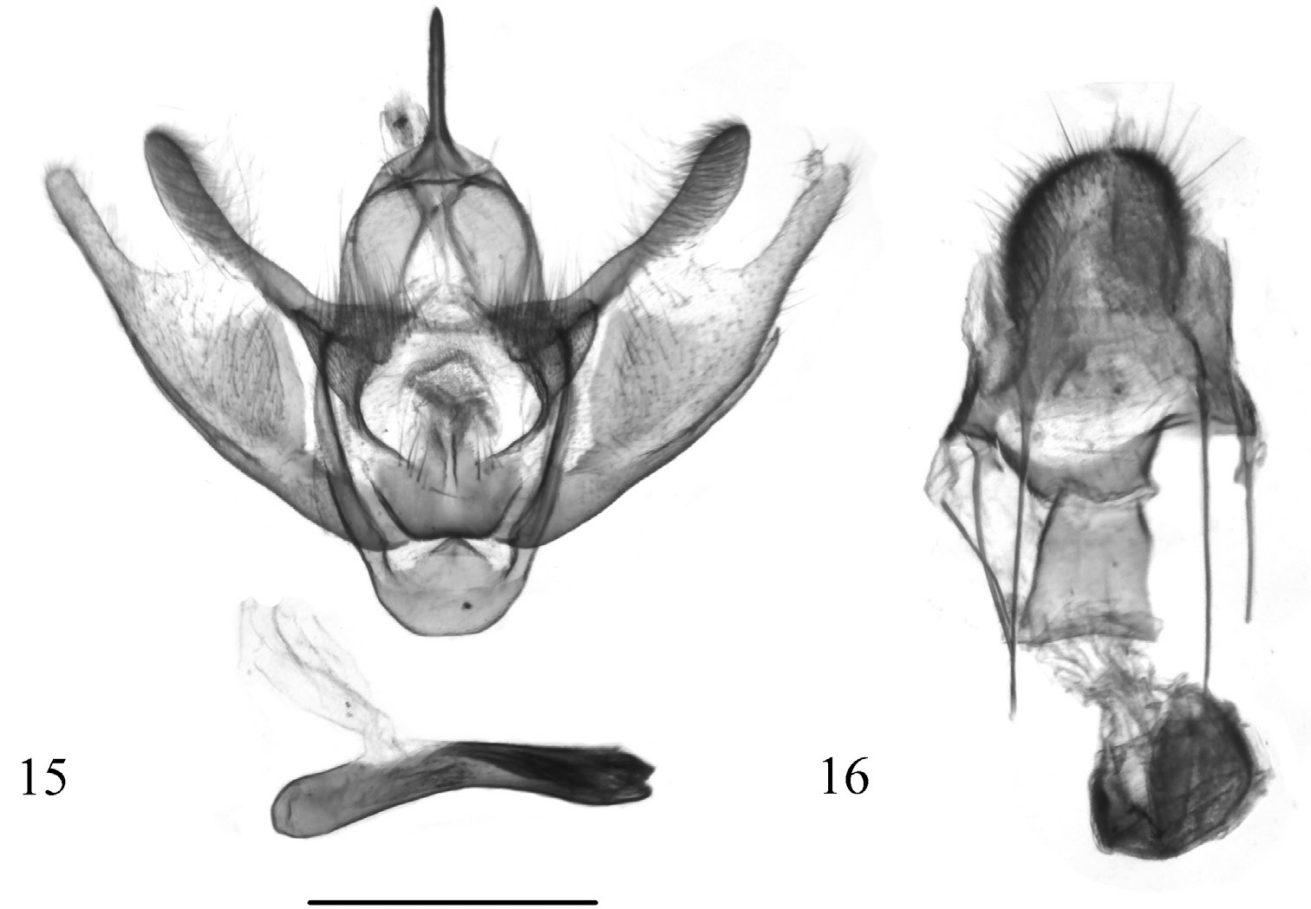
Genitalia of *Fueguinaaraucana* Ramos-González & Parra, sp. n. **15** male genitalia (holotype, MZUC-UCCC, slide No. AMLP 0139) **16** female genitalia (allotype, MZUC-UCCC, slide No. AMLP 0138). Scale bar: 1 mm.

Figures 7 , 8 , 15 , 16 , 21

#### Diagnosis.

This species can be easily distinguished from *F.varians* (Butler) and *F.celovalva* Parra by its ashy forewings, crossed by dark-brown stripes, and a less-developed saccular process. Externally, it differs from *F.magallanica* Parra by its antemedial and postmedial bands, which are less angular in *F.araucana*. Can be distinguished from congeners by three other characters: the presence of disjointed subtriangular lateral processes in the juxta, the large subrounded apical indention, which extends approximately through half of valva, and having a globular corpus bursae which is short and subequal to the length of ductus bursae.

#### Description.

Male (Fig. 7). Head: antennae filiform, subapically broadened; palpi porrect, slightly tilted up covered by straight piliform dark-brown scales and 1.5 times larger than eye diameter; frons and vertex covered with imbricated flattened whitish and dark-brown scales. Thorax: patagia covered by juxtaposed flattened whitish and dark-brown scales; tegulae covered by dark-brown scales splashed with black scales, piliform scales on the posterior region. Tibial formula 0-2-4. Forewings: background color ashy; medial and Cu_1_ veins framed by three elongated blackish spots, between postmedial and subterminal bands; termen rounded, with piliform light-brown scales; basal band blackish, curved, slightly zigzagging towards the inner margin; antemedial band dark brown, sinuous, which is thinner towards the costa and inner margin than in its medial sector; postmedial band sinuous and wide, formed by two brown-orange stripes mottled with dark brown and framed with blackish-brown scales; subterminal band dark brown, diffuse, cut off on its costal third by an ashy apical spot; discal spot present and blackish. Hindwings: same size as in females, subrectangular, ashy-brown, with a digitiform lobe extended over the base of anal margin; discal spot not visible. Wing venation (Fig. 21): forewing with two accessory cells; hindwing with Sc+R_1_ and Rs connected by a transverse vein towards one-third before the end of the cell; Rs and M_1_ pedunculated; M_2_ absent and M_3_ near Cu_1_; Cu_1_ is near the angle of the cell; Cu_2_ weak, one-fifth before the angle of the cell; lobe crossed by straight A_1_ and slightly curved A_2_; discal cell polygonal and extend for half of wing surface. Male genitalia (Fig. 15): valvae subrectangular, costa strongly sclerotized with rounded and setose apex, deep subrectangular apical notch, approximately half the length of valvae; cucullus projected in the apex of anterior edge, sacculus present and spine-like; saccus rounded; juxta with subquadrangular base and M-shaped posterior apex, with two disjointed lateral processes that have setose subtriangular apex and extends at the height of transtilla; uncus glabrous and straight. Aedeagus tubular; vesica armed with three cornutus. Female (Fig. 8): similar to males, but with filiform antennae slighter and subrectangular hindwings without lobe on the anal margin. Female genitalia (Fig. 16): ductus bursae striated and subequal in length to corpus bursae; corpus bursae globular, membranous; cestum present, subrectangular and strongly sclerotized; posterior apophyses longer than anterior ones.

#### Type material.

Holotype: 1 ♂, pinned, Chile, Araucanía, Malleco, R.N. Malalcahuello-Nalcas, Corralco, 09-XII-2014, leg. L.E. Parra, “AMLP 0139” [genitalia slide], “UCCC_MZUC_Lep_0031” [ID code], “Holotype *Fueguinaaraucana*” [red handwritten label] (MZUC-UCCC); Allotype: 1 ♀, pinned, Chile, Malleco, Río Blanco III-1951, leg. L.E. Peña, “Especie 23 H” [ID code, female], “AMLP 0138” [genitalia slide], “Allotype *Fueguinaaraucana*” [red handwritten label] (MZUC-UCCC).

Paratypes: 1 male, 3 females. Chile: **Malleco**: Curacautín, Termas de Río Blanco, 1050-1300 m, 21/24-II-1954, leg. L.E. Peña (1 ♀) (MZUC-UCCC). **Cautín**: Pucón, Termas de Río Blanco, II-1951, leg. L.E. Peña, “Especie 23 M” [ID code, male], “AMLP 0093” [wing slide] (1 ♂, 1 ♀) (MZUC-UCCC); Pucón, Termas de Río Blanco, III-1951, leg. L.E. Peña (1 ♀) (MZUC-UCCC).

#### Distribution.

This species occurs between Malleco and Cautín provinces. It is distributed in parts of Maule and Valdivian Forest biogeographic provinces, Subantarctic subregion, Andean region.

#### Flight period.

Specimens were captured in December, February and March.

**Etymology.** The species name is dedicated to the Araucanía region, Chile, the locality where all specimens were collected.

**Figures 17–21.**
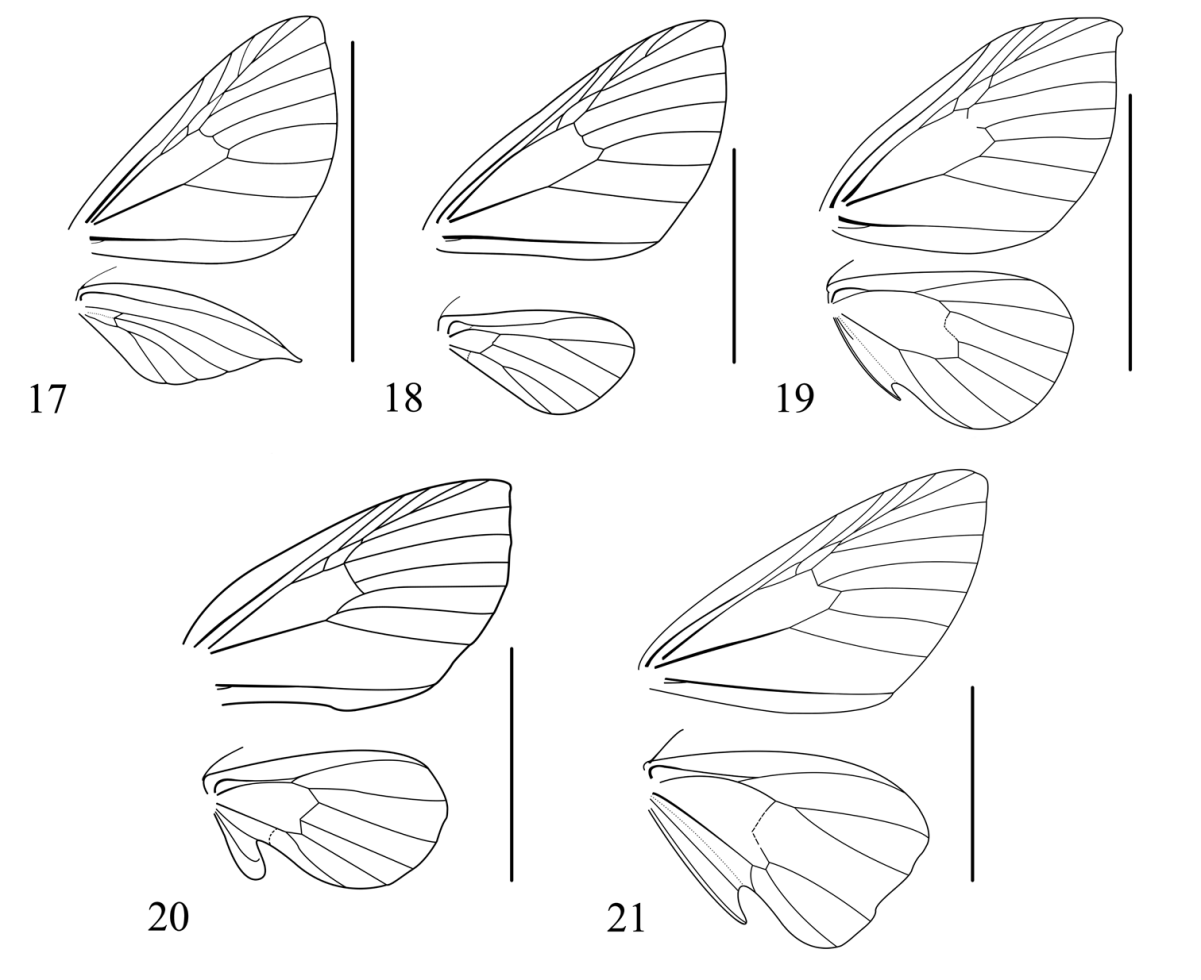
Wing venation of males **17***Alobacarolinae* Ramos-González & Parra, sp. n. **18***Hoplosaurismorenoi* Ramos-González & Parra, sp. n. **19***Butlerianaphoenix* Ramos-González & Parra, sp. n. **20***Warrenariaonca* Ramos-González & Parra, sp. n. **21***Fueguinaaraucana* Ramos-González & Parra, sp. n. Scale bar: 1 cm

## Discussion

The genus *Hoplosauris* was proposed by Butler (1882) and currently is the most species-rich Chilean trichopterygine genus with eight valid species (Parra et al. 2009, 2017). The species are: *H.granitata* (Fletcher, 1953), *H.heliconoides*Butler (1882), *H.indistincta* (Butler, 1882), *H.macarenae* Parra (2009), *H.mabillei* Parra (2009), *H.pachrophylloides* Parra (2009), *H.schausi* (Warren, 1908), and *H.valeria*Butler (1893). The genus can be recognized by three synapomorphies: a small flap, vesicle and/or tuft of piliform scales in the anal margin of the hindwing in males; a setal tuft in the subapical region of valvae; and microspines and striated areas in the internal surface if corpus bursae (Parra et al. 2009, 2017).

It is possible to include *H.morenoi* in this genus, due to the low genetic distance between this species and *H.pachrophylloides* (< 8%; Hausmann and Hebert 2009; Hausmann et al. 2011) and also because of the large number of characters shared with *H.heliconoides*, the type species. Some of these characters are the presence of short and porrect palpi; the connection of the Sc+R_1_ and Rs veins by a weak transverse vein; and the pedunculated M_3_ and Cu_1_ veins; the absence of Cu_2_ and anal veins; the presence of an anal cell; the short and triangular discal cell in the forewings of males; the valvae with brush-like subapical setal tuft and apically projected cucullus; the ductus bursae which is one-sixth the length of corpus bursae; and the subpyriform completely sclerotized corpus bursae with longitudinal striation and rows of microspines. Thus, the number of species belonging to *Hoplosauris* increases to nine.

Several Chilean Trichopterygini (e.g., *Butleriana*, *Warrenaria*, *Fueguina*, *Tomopteryx* Philippi, *Triptila* Warren, *Triptiloides* Parra & Santos-Salas, *Pachrophylla* Blanchard, and *Parapachrophylla* Parra) share ancestral characters in the male genitalia, e.g. valvae with indented posterior apex and juxta with a pair of lateral processes joined each other at transtilla height (Viidalepp 2011). This means that the venation pattern of the hindwings is particularly important for the determination of Chilean genera, especially in males (Parra 1991, 1996; Parra and Santos-Salas 1991, 1992).

Males of the genera *Butleriana* and *Llampidken* Parra have in common the shape of the lobes on the hindwing. However, venation of lobes is different in these genera, as well as some structures in the male genitalia (e.g., hooked socius, presence of saccular processes and costal arm in *Llampidken*). *Butleriana* is characterized by the presence of a single anal vein (A_2_) crossing the lobe (a synapomorphy that defines *Butleriana*). A_1_, when present, is only a remnant vein, slightly visible at the base of the hindwing. This is different in *Llampidken* in which no anal veins go across the lobe (an autapomorphy) (Parra and Santos-Salas 1992; Parra et al. 2017). Although the genetic divergence between *B.phoenix* and the type species (*B.minor*) is high (approximately 11%), it is possible to assign *B.phoenix* to the genus *Butleriana* because of the consistency in males of the hindwing and genital morphology, i.e., both species have similar valvae and a single anal vein through the subtriangular lobe, with the A_1_ vein slightly visible at its base.

It is possible to distinguish *Warrenaria* by its reddish-brown coloration, rectangular valvae, and the shape of the uncus (Parra et al. 2017). *Warrenariaonca* is included in this genus because it shares with *W.martha* (type species) the maculation and wing venation general patterns. Other similarities are: the length of apical indention in the valvae, the shape of valva and socius, and the U-shaped juxta. All these characters, combined, are unique of *Warrenaria* and do not occur in other Chilean Trichopterygini.

*Fueguina* comprises three species: *F.varians*, *F.celovalva*, and *F.magallanica*. This genus can be distinguished by the presence of three features in males: a lobe at the hindwing base with two anal veins, a spiniform saccular process, and a deep indention on the cucullus region (Parra 1991; Parra et al. 2017). It is possible to include *F.araucana* in this taxon because of the shape and venation of hindwing lobe, the presence of a costal process, and the presence of a spiniform saccular process with a deep apical indention. *Fueguinaaraucana* and *F.magallanica* share the following characters: general wing venation pattern, general shape of valvae and juxta, and the similar maculation pattern. However, there are distinctive characters in *F.araucana*: distinctive lateral processes of juxta; a deeper apical indention of valvae (in this sense, similar to *F.varians* and *F.celovalva* but more rounded, as in *F.magallanica*); and the shape of bursa copulatrix. The large number of common characters between *F.magallanica* and *F.araucana* suggests that both species are closely related, placing them as the sister-species.

Regarding *Aloba*, Warren (1895) was the first to placed this genus in the tribe Trichopterygini and included only one species. Nevertheless, this species was not considered to belong to the Trichopterygini in later works (e.g., Parra et al. 2017). After analyzing the anatomical features of this species, it is possible to recognize it as a member of the Trichopterygini and re-assign it to the tribe. This species shares with the Trichopterygini the diagnostic characters that defines the tribe, such as the reduction in the anal margin of male hindwings with subsequent simplification of venation and the presence of a sternal pouch in the tympanic opening (Dugdale 1980; Parra et al. 2017). *Aloba* can be considered as the taxon morphologically closest to *Hoplosauris*, based on the absence of lobe and anal veins in the hindwings of males, the presence of a cucullus projected apically, and a setal tuft. This taxonomic relationship is supported by molecular phylogenetic analyses (Ramos-González et al. unpublished data; Brehm et al. submitted).

Finally, considering all these new findings, the number of Chilean Trichopterygini increases to 15 genera and 45 species.

## Supplementary Material

XML Treatment for Aloba

XML Treatment for Aloba carolinae

XML Treatment for Hoplosauris morenoi

XML Treatment for Butleriana phoenix

XML Treatment for Warrenaria onca

XML Treatment for Fueguina araucana
